# Lateral Antebrachial Cutaneous Neuropathy: A Review of 15 Cases

**DOI:** 10.7759/cureus.25203

**Published:** 2022-05-22

**Authors:** Anza B Memon, Selina Mahmood, Furqan Waseem, Frederick Sherburn, Ashley Nardone, Bashiruddin K Ahmad

**Affiliations:** 1 Neurology, Henry Ford Health System, Detroit, USA; 2 Neurology, Wayne State University School of Medicine, Detroit, USA

**Keywords:** lateral antebrachial cutaneous neuropathy (labcn), forearm trauma, phlebotomy, orthopedic procedure, forearm numbness

## Abstract

Background: Lateral antebrachial cutaneous nerve is a terminal sensory branch of the musculocutaneous nerve. Lateral antebrachial cutaneous neuropathy (LABCN) is rare and often underdiagnosed. Less than 100 cases have been described in the orthopedic literature.

Methods: It’s a single-center retrospective study. A retrospective chart review of patients with LABCN who were seen over 16 years was performed. Demographics and detailed clinical information were recorded. In addition, electrodiagnostic data were reviewed, and clinical outcome was recorded.

Results: Fifteen patients were included in this study. Postsurgical etiology was the most common (n = 7) cause of LABCN. Other cases included antecubital fossa phlebotomy and intravenous placement (n = 4), trauma (n = 1), overuse or repetitive forearm use (n = 2), and dog bite (n = 1). No etiology was found in one case, but the patient had diabetes.

Conclusion: Our study proposes that patient positioning during orthopedic surgeries leading to stretch or compression of the lateral antebrachial cutaneous nerve is the most likely cause of LABCN. Antecubital fossa needle placement is the second most common cause of LABCN. However, it’s a rare mononeuropathy and can be underdiagnosed. Therefore, detailed history, examination, and nerve conduction studies of the bilateral lateral antebrachial cutaneous nerve could help establish the diagnosis after other etiologies have been carefully excluded.

## Introduction

The lateral antebrachial cutaneous nerve is a terminal sensory branch of the musculocutaneous nerve. It provides sensory innervation to the lateral or radial half of the forearm. Lateral antebrachial cutaneous neuropathy (LABCN) is rare, and less than 100 cases have been described in the orthopedic and sports medicine literature [1]. Lateral forearm paresthesia without motor deficits is common [2,3]. The nerve lies deep in the distal biceps muscle and its tendon. It pierces the deep fascia just proximal to the elbow joint. It then passes behind the cephalic vein and divides into dorsal and volar branches [4,5]. Detailed history and neurological examination are the keys to the definitive diagnosis. However, electrodiagnostic studies such as nerve conduction and electromyography are often required to exclude other mimics such as brachial plexopathy. Common causes of LABCN include venipuncture, direct trauma to the elbow, and prolonged compression at the elbow or forearm segment [6,7].

This study aimed to understand the clinical presentation and etiology of LABCN. The secondary purpose was to understand the electrophysiological findings of LABCN and its role in diagnosis and prognostication. This article was previously presented as a meeting abstract at the 2019 AANEM, American Association of Neuromuscular & Electrodiagnostic Medicine.

## Materials and methods

Study design

Patients seen at our health system between January 2000 and December 2016 with an electromyography diagnostic code of LABCN were identified via the electronic medical record. The Henry Ford Hospital Institutional Review Board (IRB # 13014) approved the study. Institutional IRB waived the need for informed consent given the study’s retrospective design. A retrospective chart review of patients with LABCN diagnosis was performed.

Inclusion criteria

Patient age at onset of symptoms, gender, side of nerve injury, duration of symptoms, etiology, and surgical history was recorded. In addition, electrophysiologic studies (EPS) and recovery data were collected. Antidromic stimulation of the lateral antebrachial cutaneous nerve was performed using Spindler and Felsenthal’s technique [8]. All patients underwent EPS. EPS confirmed prolonged distal latency or reduced amplitude on the symptomatic side compared with the contralateral side.

Exclusion criteria

Patients with brachial plexopathies and motor weakness in the biceps and brachialis muscles to suggest musculocutaneous neuropathy were excluded from the study.

Statistical analysis

Statistical analysis was not needed due to the study’s small sample size and descriptive design. For the same reason, a comparison among different study parameters cannot be performed.

## Results

Of 15 patients included in this study, seven were male and eight were female. The mean age at the diagnosis was 53 years old (range 36-82). Postsurgical etiology was the most common (n = 7) cause of LABCN during orthopedic surgeries. Other cases included antecubital fossa phlebotomy and intravenous placement (n = 4), trauma (n = 1), repetitive forearm use (n = 2), and dog bite (n = 1). No etiology was found in one case, but the patient had diabetes.

Two of the seven postsurgical patients had direct surgical trauma. However, five patients developed symptoms secondary to arm positioning during orthopedic procedures such as shoulder (n = 4) and knee surgeries (n = 1).

Lateral antebrachial cutaneous nerve conductions were performed on all patients included in the study. Thirteen patients showed absent or reduced sensory amplitude, and only two showed a demyelinating pattern with prolonged sensory distal latencies on EPS. Sensory responses were absent in 7/13 patients with an axonal neuropathy pattern, which was deemed severe. Eight patients had poor clinical outcomes, three had a good recovery, and four patients were lost to follow-up. None of the patients had repeat lateral antebrachial cutaneous nerve conductions. Demographic, clinical, and electrodiagnostic findings are listed in Table 1.

**Table 1:** Demographics, clinical characteristics, and electrodiagnostic findings of lateral antebrachial cutaneous neuropathy A: axonal; BL: bilateral; CTS: carpal tunnel syndrome; D: demyelinating; Dx: diagnosis; EMG: electromyography; F: female; IV: intravenous; L: left; M: male; neuro: neurology; NR: no response; NS: neurosurgery; ortho: orthopedics; R: right; radic: radiculopathy; SLAP: superior labrum anterior and posterior

Patient No.	Sex	Age (years)	Referral	Side	Reason for EMG	Symptoms duration (months)	Etiology	Surgery	Diabetes	Other coexisting EMG Dx?	Type of nerve injury	Prognosis
1	M	46	Ortho	L	Pain	48	Nonspecific arm trauma	Radial tunnel release surgery x 2	No	Mild chronic left C8-T1 radic	A	Poor
2	F	54	NS	R	Numbness/pain	12	Postsurgical	Rotator cuff surgery	No	Right medial antebrachial neuropathy	D	Poor
3	M	42	Ortho	R	Numbness/pain	12	Postsurgical	SLAP repair	No	Mild remote C8	A (severe)	Poor
4	F	77	Ortho	R	Numbness/pain	8	Postsurgical	Right hemiarthroplasty	No	Mild CTS/mild cervical radic chronic	A (severe)	Poor
5	M	43	Ortho	L	Numbness	Unknown	Trauma		No		A (severe)	Unknown
6	F	52	Neuro	L	Numbness/pain	1	Repetitive forearm use (clothing competition)		No		A (severe)	Unknown
7	F	57	Plastic surgery	L	Tingling	48	Repetitive forearm use (gardening)		No	Left medial antebrachial neuropathy	A	Good
8	F	82	Neuro	L	Numbness/pain	4	Unknown		Yes	BL CTS/cervical radic	D	Poor
9	M	42	Ortho	L	Numbness/pain	5	Postsurgical	Resection of left radial head prosthesis/forearm incision	No	Suprascapular; remote left C8	A (severe)	Good
10	F	60	Neuro	L	Pain	2	Blood draw antecubital fossa		No	Superficial radial sensory	A	Good
11	M	72	Ortho	R	Numbness/tingling	48	Postsurgical	Rotator cuff surgery for shoulder trauma from fall	No	Musculocutaneous (secondary to fall and shoulder injury)	A	Poor
12	M	36	Medicine	R	Numbness/pain	24	Blood draw antecubital fossa		No		A	Poor
13	F	37	NS	R	Numbness	4	Dog bite		No		A	Poor
14	F	42	Neuro	L	Pain/tingling	8	Postsurgical	Cubital tunnel, pronator release and trigger release of the thumb	Yes		A (severe)	Unknown
15	M	61	Medicine	R	Numbness	2	IV line in antecubital fossa		Yes	BL CTS	A (severe)	Unknown

None of the patients had repeat lateral antebrachial cutaneous nerve conductions. Demographic, clinical, and electrodiagnostic findings are listed in Table 1.

## Discussion

LABCN is rare and often misdiagnosed. Most of the cases are reported in the orthopedic literature. Common differential diagnoses of LABCN include cervical radiculopathy, lateral epicondylitis, pronator teres syndrome, radial tunnel, biceps tendonitis, brachial plexopathy, and neuralgic amyotrophy, and median and radial neuropathy [1,9]. Commonly reported etiologies are repetitive forceful pronation in throwing athletes, compression due to a tourniquet, antecubital fossa phlebotomy, improperly placed blood pressure cuff, restraining strap, and positioning while under general anesthesia [1]. In some case series, direct compression of the nerve by carrying heavy trays in waitresses and sustained elbow flexion during windsurfing (board sailing) have also been reported [7,10]. Diagnosis of LABCN can be established based on the patient’s history, physical exam, and nerve conduction studies (NCS) [2,3].

In our case series, we elaborate on some of the common causes of LABCN. There have been few case reports and at least one case series on LABCN that we could find in the literature [7]. In our study, we report five patients who developed LABCN secondary to arm positioning during orthopedic surgeries. A similar case is reported in the literature in a patient who underwent a meniscal transplant and microfracture surgery due to positioning while under general anesthesia [1]. American Society of Anesthesiologists Task Force on Preventing Perioperative Peripheral Neuropathies suggests that the ideal surgical position should include minimal shoulder extension, external rotation, abduction, and slight elbow flexion with forearm supination with a neutral wrist position [11,12]. This position is suggested after thorough preoperative history and examination are performed [11,12]. Our study demonstrated that the sensory axon loss on NCS did not correlate with patients’ clinical recovery. However, this should be interpreted with caution since none of the patients in our study cohort had repeat NCS.

## Conclusions

In conclusion, this study demonstrates some of the most common causes of LABCN. It also demonstrated the risk of LABCN during orthopedic procedures that could be secondary to arm positioning during general anesthesia. Careful history and examination can help guide clinicians in diagnosing this under-recognized cause of mononeuropathy. Our study showed that the axon loss on the lateral antebrachial cutaneous nerve conductions does not correlate with the clinical outcome. However, one must be careful to comment on that part, given a lack of follow-up studies. Retrospective design and small sample size are the limitations of our study.
